# Childhood maltreatment and health in the UK Biobank: triangulation of outcome-wide and polygenic risk score analyses

**DOI:** 10.1186/s12916-024-03360-9

**Published:** 2024-03-25

**Authors:** Ana Lucia Espinosa Dice, Rebecca B. Lawn, Andrew Ratanatharathorn, Andrea L. Roberts, Christy A. Denckla, Ariel H. Kim, Pedro A. de la Rosa, Yiwen Zhu, Tyler J. VanderWeele, Karestan C. Koenen

**Affiliations:** 1 Department of Epidemiology, Harvard T.H. Chan School of Public Health, Boston, MA USA; 2 Stanley Center for Psychiatric Research, Broad Institute of MIT and Harvard, Cambridge, MA USA; 3 Department of Epidemiology, Columbia University Mailman School of Public Health, New York City, NY USA; 4 Department of Environmental Health, Harvard T.H. Chan School of Public Health, Boston, MA USA; 5 Department of Social and Behavioral Sciences, Harvard T.H. Chan School of Public Health, Boston, MA USA; 6 Department of Molecular and Cellular Biology, Harvard University, Cambridge, MA USA; 7 Institute for Culture and Society, University of Navarra, Pamplona, Spain; 8 Department of Biostatistics, Harvard T.H. Chan School of Public Health, Boston, MA USA; 9 Psychiatric Neurodevelopmental Genetics Unit, Department of Psychiatry, Massachusetts General Hospital, Boston, MA USA

**Keywords:** Childhood maltreatment, UK Biobank, Triangulation, Outcome-wide analysis, Polygenic risk score

## Abstract

**Background:**

Childhood maltreatment is common globally and impacts morbidity, mortality, and well-being. Our understanding of its impact is constrained by key substantive and methodological limitations of extant research, including understudied physical health outcomes and bias due to unmeasured confounding. We address these limitations through a large-scale outcome-wide triangulation study.

**Methods:**

We performed two outcome-wide analyses (OWAs) in the UK Biobank. First, we examined the relationship between self-reported maltreatment exposure (number of maltreatment types, via Childhood Trauma Screener) and 414 outcomes in a sub-sample of 157,316 individuals using generalized linear models (“observational OWA”). Outcomes covered a broad range of health themes including health behaviors, cardiovascular disease, digestive health, socioeconomic status, and pain. Second, we examined the relationship between a polygenic risk score for maltreatment and 298 outcomes in a non-overlapping sample of 243,006 individuals (“genetic OWA”). We triangulated results across OWAs based on differing sources of bias.

**Results:**

Overall, 23.8% of the analytic sample for the observational OWA reported at least one maltreatment type. Of 298 outcomes examined in both OWAs, 25% were significant in both OWAs and concordant in the direction of association. Most of these were considered robust in the observational OWA according to sensitivity analyses and included outcomes such as marital separation (OR from observational OWA, OR_*o*_ = 1.25 (95% CI: 1.21, 1.29); OR from genetic OWA, OR_*g*_ = 1.06 (1.03, 1.08)), major diet changes due to illness (OR_*o*_ = 1.27 (1.24, 1.29); OR_*g*_ = 1.01 (1.00, 1.03)), certain intestinal diseases (OR_*o*_ = 1.14 (1.10, 1.18); OR_*g*_ = 1.03 (1.01, 1.06)), hearing difficulty with background noise (OR_*o*_ = 1.11 (1.11, 1.12); OR_*g*_ = 1.01 (1.00, 1.01)), knee arthrosis (OR_*o*_ = 1.13 (1.09, 1.18); OR_*g*_ = 1.03 (1.01, 1.05)), frequent sleeplessness (OR_*o*_ = 1.21 (1.20, 1.23); OR_*g*_ = 1.02 (1.01, 1.03)), and low household income (OR_*o*_ = 1.28 (1.26, 1.31); OR_*g*_ = 1.02 (1.01, 1.03)). Approximately 62% of results were significant in the observational OWA but not the genetic OWA, including numerous cardiovascular outcomes. Only 6 outcomes were significant in the genetic OWA and null in the observational OWA; these included diastolic blood pressure and glaucoma. No outcomes were statistically significant in opposite directions in the two analyses, and 11% were not significant in either OWA.

**Conclusions:**

Our findings underscore the far-reaching negative effects of childhood maltreatment in later life and the utility of an outcome-wide triangulation design with sensitivity analyses for improving causal inference.

**Supplementary Information:**

The online version contains supplementary material available at 10.1186/s12916-024-03360-9.

## Background

Childhood maltreatment, which includes emotional abuse and neglect, physical abuse and neglect, and sexual abuse, is common globally, with prevalence ranging from 15 to 25% [1, 2]. Childhood maltreatment is associated with substantial morbidity and mortality [3, 4] across domains of physical [5–9] and psychosocial health [10–18] and with worse socioeconomic status [19]. Despite extensive literature documenting a connection between childhood maltreatment and poorer health and well-being, our understanding of the impact of maltreatment in later life is constrained by key substantive and methodological limitations of extant research.

Regarding substantive issues, the relationship of childhood maltreatment with several domains of physical health remains poorly understood. For example, despite the association of childhood maltreatment with ocular [20–23] and dental [24–28] health in childhood and increasing recognition of the link between these health outcomes and stress- and inflammation-related disorders [29, 30], the long-term sequelae of childhood maltreatment in these domains are not well documented [31–33]. Furthermore, findings remain inconclusive for some key physical health outcomes, including blood pressure [34–39] and chronic pain [40, 41]. In addition, varying approaches to selection and grouping of outcomes limit our understanding of the broad impact of maltreatment, the comparability of findings, and the interpretability of results. Prior work has typically examined just one or a few selected outcomes, which impedes comparison of findings across outcomes and precludes identification of unexpected sequelae of maltreatment. In addition, some prior work has grouped outcomes into broad or inconsistent categories [42, 43], which may conflate outcomes with diverse etiologies and reduce the ability to disentangle potential bio-behavioral pathways linking childhood maltreatment to health.

Regarding methodological issues, evidence suggests that results linking maltreatment to adverse outcomes may be inflated by recall bias in the reporting of maltreatment [44, 45] as well as confounding by childhood poverty and related neighborhood factors [19, 46, 47]. Discussions of recall bias in the literature have focused largely on the advantages of using prospective measures of maltreatment and reports from multiple informants, though these alternatives to retrospective self-reports may underestimate certain types of maltreatment and fail to capture key mechanisms linking maltreatment and mental health [44, 48–50]. Less examined are techniques such as *E*-values, which have been used to assess robustness of observational results to unmeasured confounding in the epidemiologic literature [51–53] but have not been widely used in studies of maltreatment. A relatively new approach is the use of genetic data as a surrogate for exposure, given that genetic markers are affected by types of biases (e.g., weak instrument, population stratification, pleiotropy) that are different from the sources of bias that commonly affect observational studies and produce non-causal results (e.g., residual confounding, reverse causation, information bias) [54–56]. To date, the use of genetic data as a proxy for childhood maltreatment has been limited [56–60].

In the present study, we address the substantive limitations by estimating associations between self-reported childhood maltreatment and over 400 adulthood social, economic, and health indicators, including both leading causes of mortality and diverse markers of quality of life. This outcome-wide analysis (OWA) approach aims to: reduce potential investigator bias in outcome selection, facilitate comparison of effect sizes, transparently report null results, use consistent confounding control, correct for multiple testing, and examine robustness of results to unmeasured confounding [61, 62]. We address other methodological issues by repeating the OWA using a polygenic risk score (PRS) for childhood maltreatment, which was derived using both prospective and retrospective data on childhood maltreatment. This PRS is less susceptible to recall bias given the high degree of shared genetic variance between the PRS from prospective and the PRS from retrospective reports [56]. A PRS can be used as a proxy for an exposure based on independent genetic variants and is less susceptible to reverse causation or residual confounding from childhood experiences [56, 63, 64]. Finally, we triangulate across these two OWAs and examine the concordance of results across measures of childhood maltreatment. Given that each OWA is influenced by different types of confounding and measurement bias, the comparison and integration of both sets of results presents an opportunity for more reliable causal inference regarding the effects of maltreatment in later life [55]. Our triangulation approach using genetic data, which to our knowledge has not been applied to the study of maltreatment, allows us to increase our confidence in a causal association when results are concordant.

## Methods

### Methods overview

First, we performed an OWA on an analytic sample of 157,316 UK Biobank participants with maltreatment data, examining the relationship of self-reported maltreatment with 414 outcomes. Second, we investigated the relationship between PRS for maltreatment and our outcomes in a sample of 243,006 participants with genetic data but no maltreatment data. For simplicity, we refer to analyses using self-reported maltreatment exposure as the observational OWA and analyses using maltreatment PRS as the genetic OWA.

### Study design and participants

UK Biobank is a longitudinal population-based study that enrolled over 500,000 participants aged 40–69 years between 2006 and 2010. In 2016, 339,092 subjects with a known email address were invited to complete a web-based Mental Health Questionnaire, which included questions about their experiences of childhood maltreatment [65]. In total, 157,366 (46%) responded by July 2017, the end of follow-up. After removing withdrawn participants (*n* = 19 from this sub-sample) and those who responded to the Mental Health Questionnaire but not to any questions about maltreatment (*n* = 31 from this sub-sample), we performed an observational OWA on 157,316 participants who responded to at least one maltreatment question.

Quality control (QC) procedures for UK Biobank genetic data have been described [66, 67]. A total of 361,194 unrelated participants of European genetic ancestry had genetic data. Participants of European ancestry with both genetic and maltreatment data were included in a prior meta-GWAS of maltreatment (185,414 participants total across 5 cohorts, using retrospective self-report data from UK Biobank and PGC_26K and prospective reports of maltreatment made largely by parents/caregivers from ABCD, ALSPAC, and Generation R; see meta-GWAS study for a very thoughtful discussion of the implications of studying the genetics of maltreatment as well) [56]. In the present study, we derived maltreatment PRS in participants with genetic data but no maltreatment data, using summary statistics from the prior meta-GWAS [56, 68]. We included only individuals of European ancestry due to the poor predictive performance of existing PRS (derived from Eurocentric GWAS) in non-European ancestry populations [69, 70] and the relative lack of power to study individuals of non-European ancestry in UK Biobank [71, 72]. Our final analytic sample for the genetic OWA thus included 243,006 individuals. There was no overlap between analytic samples for observational and genetic OWAs.

### Measures

#### Childhood maltreatment

The Childhood Trauma Screener [73] consists of five items describing experiences of each of the following five trauma types, respectively: emotional abuse and neglect, physical abuse and neglect, and sexual abuse. Response options ranged from “never true” to “very often true” on a 5-point scale. Participants also could indicate “prefer not to say.” We dichotomized each type of maltreatment at validated cut-offs [74] and created a categorical count variable for the number of types of maltreatment: 0 (reference), 1, 2, 3, and 4–5 (combined due to low prevalence in the highest category). Maltreatment questionnaire items and coding are detailed in Additional file 1: Table S1.

#### Outcomes

To select outcomes, we used results from a prior factor analysis of 730 variables in the UK Biobank [66]. Variables were selected from a set of 3011 unique phenotypes derived using a modified version of the PHEnome Scan ANalysis Tool [75] in addition to 633 ICD-10 codes at the three-digit category level (i.e., capturing the category of diagnosis without specific details about the etiology or anatomic site). Variables were included if they had prevalence (> 1%) and were excluded if they exhibited high collinearity or structured/item-dependent missingness. There were 505 variables that loaded onto 35 latent factors [66]. Here, we then excluded 80 variables with > 40% missingness and 11 that were about childhood (due to temporality concerns: sunburns before age 15, comparative body size to others at age 10, comparative height at age 10) or that we already used as covariates (maternal smoking at birth, maternal severe depression, number of siblings, 5 maltreatment questions).

**Fig. 1:**
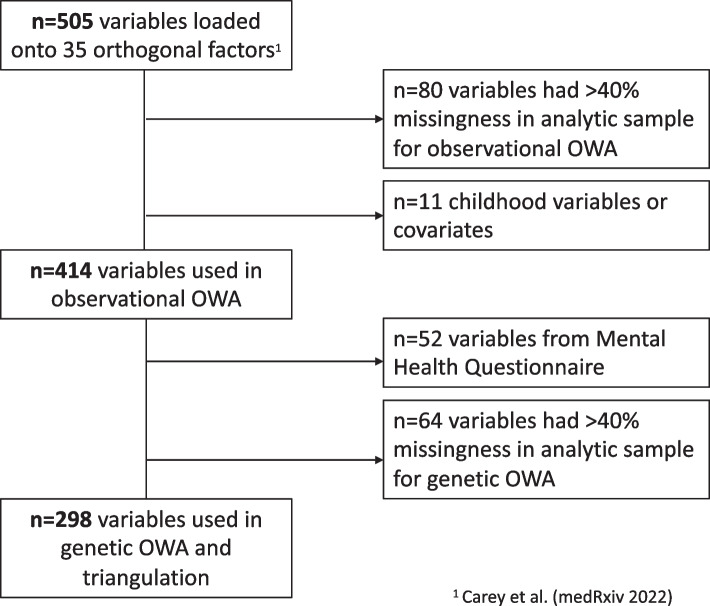
Flow chart of study outcomes selection process

We included 414 adulthood outcomes in our observational OWA. These outcomes included 79 continuous, 331 binary, and 4 categorical variables related to the following 18 themes (in decreasing order by number of outcomes): trauma and mental illness; work and workplace; diet and supplements; home environment and transport; alcohol, drugs, sex, and lifestyle; joints and pain; cardiovascular and diabetes; digestive, bowel, and abdomen; respiratory; physical activity; inflammation, cancer, and blood; sleep; general health; education, income, and finances; body composition and weight; skin, mouth, and teeth; hearing and eyes; and reaction time. Outcomes were based on a mix of self-report, medical record, geo-code, physical assessment, and biological sampling data. See Fig. 1 for a flow chart of the outcome selection process and Additional file 1: Table S2 for further details on study outcomes.

For the genetic OWA, we included 298 of the 414 outcomes used in the observational OWA. As our sample for the genetic OWA was chosen from participants who did not complete the Mental Health Questionnaire, 52 outcomes derived from this questionnaire were not available. We excluded 64 additional outcomes as > 40% of participants in our analytic sample were missing these measures; most of these outcomes were from the web-based Work Environment Follow-Up Questionnaire, which included a largely overlapping sample with that of the Mental Health Questionnaire (both questionnaires required a known email address on file).

#### Observational OWA covariates and missingness

Covariates included sociodemographic, childhood, and family factors assessed retrospectively. Sociodemographic covariates were sex, country of birth, ethnicity, age at enrollment, and age at the Mental Health Questionnaire. Country of birth was classified as the UK (England, Scotland, Wales, Northern Ireland) versus elsewhere (including Ireland). Ethnicity was classified as white, Mixed, Asian, Black, or another race, combining response options of Asian and Chinese to match the 2011 Census of England and Wales [76]. Childhood factors were participant-reported and included birthweight, number of siblings, and whether they were breastfed (yes/no) and a twin or multiple (yes/no). Family factors included whether the participant’s mother smoked around birth (yes/no) and ever suffered from severe depression (yes/no). All confounders, excluding age at completion of the Mental Health Questionnaire, were assessed at enrollment (2006–2010). When a response was missing (or “prefer not to say” and “do not know”) at enrollment, responses were imputed from follow-up instances of the same question when possible. Otherwise, these missing values were multiply imputed.

Specifically, we used multiple imputation with chained equations (*m* = 10; max iterations = 10) [77] to impute covariates as well as “prefer not to say” responses to the maltreatment questions (< 1% prevalence for each maltreatment type). Numeric variables were imputed with predictive mean matching; binary and categorical variables were imputed with logistic and polytomous regression, respectively. We could not include all 414 outcomes in the imputation model due to model convergence issues. Instead, we included the top three unique outcomes within each of the 35 latent factors based on the magnitude of item loadings [66]. See Additional file 1: Tables S3A–C as well as Table 1 for further details on auxiliary variables, the imputation of maltreatment “prefer not to say” responses, and amount of missingness in covariates.

**Table 1:** Observational outcome-wide analysis analytic sample descriptive characteristics

Covariate	Categorical count of maltreatment types					
	**0 (*N* = 119,893)**	**1 (*N* = 25,030)**	**2 (*N* = 7924)**	**3 (*N* = 3150)**	**4–5 (*N* = 1319)**	**Total (*N* = 157,316)**
**Sex**						
Female	65,921 (55.0%)	14,989 (59.9%)	5044 (63.7%)	2135 (67.8%)	984 (74.6%)	89,073 (56.6%)
Male	53,972 (45.0%)	10,041 (40.1%)	2880 (36.3%)	1015 (32.2%)	335 (25.4%)	68,243 (43.4%)
**Maternal smoking around birth**						
*N* missing	14,823	3290	1095	461	222	19,891
No	76,842 (73.1%)	14,608 (67.2%)	4331 (63.4%)	1584 (58.9%)	572 (52.1%)	97,937 (71.3%)
Yes	28,228 (26.9%)	7132 (32.8%)	2498 (36.6%)	1105 (41.1%)	525 (47.9%)	39,488 (28.7%)
**Ethnicity**						
*N* missing	342	91	45	16	4	498
White	116,982 (97.9%)	23,864 (95.7%)	7340 (93.2%)	2889 (92.2%)	1184 (90.0%)	152,259 (97.1%)
Mixed	461 (0.4%)	188 (0.8%)	91 (1.2%)	46 (1.5%)	43 (3.3%)	829 (0.5%)
Asian	1010 (0.8%)	428 (1.7%)	174 (2.2%)	68 (2.2%)	24 (1.8%)	1704 (1.1%)
Black	573 (0.5%)	264 (1.1%)	176 (2.2%)	94 (3.0%)	42 (3.2%)	1149 (0.7%)
Other	525 (0.4%)	195 (0.8%)	98 (1.2%)	37 (1.2%)	22 (1.7%)	877 (0.6%)
**Country of birth**						
*N* missing	66	23	12	3	1	105
Not born in the UK	7674 (6.4%)	2291 (9.2%)	877 (11.1%)	361 (11.5%)	148 (11.2%)	11,351 (7.2%)
Born in the UK	112,153 (93.6%)	22,716 (90.8%)	7035 (88.9%)	2786 (88.5%)	1170 (88.8%)	145,860 (92.8%)
**Breastfed as a baby**						
*N* missing	22,721	4941	1630	714	361	30,367
No	24,931 (25.7%)	5481 (27.3%)	1965 (31.2%)	861 (35.3%)	355 (37.1%)	33,593 (26.5%)
Yes	72,241 (74.3%)	14,608 (72.7%)	4329 (68.8%)	1575 (64.7%)	603 (62.9%)	93,356 (73.5%)
**Age at enrollment**						
Mean (SD)	56.2 (7.7)	55.5 (7.8)	54.6 (7.8)	54.1 (7.7)	53.9 (7.8)	55.9 (7.7)
Range	38–72	40–70	39–72	40–70	40–70	38–72
**Age at Mental Health Questionnaire**						
Mean (SD)	63.3 (7.7)	62.6 (7.8)	61.6 (7.8)	61.2 (7.7)	60.9 (7.8)	63.0 (7.7)
Range	45–80	45–79	46–79	46–78	46–78	45–80
**Part of multiple birth**						
*N* missing	1479	415	179	91	60	2224
Single birth	116,126 (98.1%)	24,083 (97.8%)	7560 (97.6%)	2980 (97.4%)	1221 (97.0%)	151,970 (98.0%)
Multiple birth	2288 (1.9%)	532 (2.2%)	185 (2.4%)	79 (2.6%)	38 (3.0%)	3122 (2.0%)
**Number of siblings**						
*N* missing	270	85	34	16	8	413
Mean (SD)	1.9 (1.6)	2.2 (1.8)	2.5 (2.1)	2.6 (2.1)	2.9 (2.4)	2.0 (1.7)
Range	0–25	0–22	0–32	0–15	0–15	0–32
**Birthweight, kg**						
*N* missing	47,358	10,183	3184	1352	620	62,697
Mean (SD)	3.35 (0.61)	3.34 (0.64)	3.32 (0.67)	3.33 (0.67)	3.25 (0.74)	3.34 (0.62)
Range	0.48–9.00	0.45–6.35	0.91–10.00	0.68–6.46	0.88–6.35	0.45–10.00
**Maternal severe depression**						
*N* missing	3175	1001	536	264	171	5147
No	109,876 (94.1%)	21,895 (91.1%)	6533 (88.4%)	2457 (85.1%)	947 (82.5%)	141,708 (93.1%)
Yes	6842 (5.9%)	2134 (8.9%)	855 (11.6%)	429 (14.9%)	201 (17.5%)	10,461 (6.9%)

*UK* United Kingdom, *SD* standard deviation

### Statistical analysis

#### Observational outcome-wide analysis

The OWA framework proposes investigation of the relationship of a single exposure with multiple outcomes simultaneously using consistent confounding control and multiple testing correction [61, 62]. Using this framework, we fit separate models for each outcome using the categorical maltreatment count and all covariates. We standardized continuous outcomes then fit linear regression models. For binary outcomes with prevalence < 10%, we fit logistic regressions. For binary outcomes with prevalence ≥ 10%, we fit Poisson regressions with robust variance estimation [78, 79]. For categorical outcomes, we fit multinomial models instead of ordinal logistic regressions, as the proportional odds assumption was violated according to both the Brant-Wald tests and plotting. We performed multiple testing correction using false discovery rate (FDR) correction [80]; this correction based on 419 tests (414 outcomes, including some categorical) was performed separately for each level of maltreatment count versus zero.

We performed three sensitivity analyses. First, we calculated *E*-values to examine potential unmeasured confounding [51–53]. *E*-values quantify the minimum strength of association, on the risk ratio scale, that unmeasured confounder(s) would need to have with both the exposure and the outcome to fully explain away the observed exposure-outcome association, conditional on measured covariates [51]. To assess *robustness* of observed associations, we compared the magnitude of the *E*-values to the magnitude of the maternal smoking at birth coefficients (OR/RRs). A priori, we expected residual confounding from unobserved childhood poverty and neighborhood factors, given prior evidence on the topic [19, 46, 47] and the unavailability of childhood variables in the UK Biobank. However, evidence suggests that maternal smoking during pregnancy is a strong proxy for childhood socioeconomic status [81–85] and has been used as such in prior work [42]. Thus, we used the maternal smoking coefficient as a plausible estimate of the association that may be expected between an unmeasured childhood confounder and each outcome, conditional on observed covariates and childhood maltreatment. For each outcome, an association was evaluated as being potentially *robust* if the *E*-value for the lower 95% CI (i.e., a more stringent measure capturing the minimum unmeasured confounding required such that the CI could be moved to contain the null, rather than the point estimate being null) was larger in magnitude than the corresponding maternal smoking at birth coefficient estimate. When this comparison was not possible (i.e., with continuous outcomes, where the maternal smoking coefficient was a *β* estimate rather than a ratio), we considered an association to be *robust* if the *E*-value for its lower 95% CI was in the top 50th percentile of CI *E*-values of all outcomes. Second, for easier summary and visualization due to a reduced number of coefficient estimates per outcome, we performed a test of trend where we treated the 5-level categorical maltreatment count as a continuous variable. Finally, we stratified analyses by sex, exploring both the categorical maltreatment count as well as the continuous maltreatment variable for the test of trend.

#### Genetic outcome-wide analysis

We calculated PRS for childhood maltreatment using PRSice-2 [56]. Variant QC followed that of the Neale Lab’s UK Biobank mega-GWAS, with modification only to the minor allele frequency (MAF) threshold due to reduced sample size in the sub-sample [66, 67]. Specifically, autosomal single nucleotide polymorphisms (SNPs) were included if they had MAF > 0.01, Hardy Weinberg Equilibrium (HWE) *p*-value > 1e − 10, and imputation information score > 0.8. A total of 9,451,730 SNPs overlapped between the childhood maltreatment GWAS [56] and UK Biobank data. SNPs were clumped based on an *R*^2^ threshold of 0.1 and a distance threshold of 250 kb. In primary analyses, PRS was generated as the number of risk alleles weighted by GWAS [56] effect size based on genome-wide significance (*p* < 5e − 08; 12 SNPs), representing SNPs most likely to be associated with the experience of childhood maltreatment, and then standardized. We ran separate regressions of outcomes on the PRS, adjusting for birth year, sex, sequencing array, and the top 20 principal components calculated within the European ancestry sample alone. We performed multiple testing correction using false discovery rate (FDR) correction; this correction was based on 303 tests (298 outcomes, including some categorical).

We also performed three sensitivity analyses. First, although more likely to be biased due to pleiotropic effects, we created a second PRS more strongly associated with the experience of childhood maltreatment by using a less stringent *p*-value threshold (*p* < 0.5; 299,049 SNPs) [56]. Second, we re-fit analyses using categorical quintiles of each unstandardized PRS (i.e., at both *p*-value thresholds) and performed a linear test of trend where we treated the categorical quintiles as a continuous variable. Third, we stratified analyses by sex using the standardized PRS at both *p*-value thresholds.

## Results

### Descriptives

Among our observational OWA analytic sample of 157,316 participants, 56.6% were female, and 97.1% were white (Table 1, which summarizes the sample prior to imputation). Overall, 23.8% reported at least one maltreatment type. Specifically, 15.9% reported one, 5.0% reported two, 2.0% reported three, and 0.8% reported 4–5 types of maltreatment. Across maltreatment counts, emotional abuse (9.5%) was the most frequently reported type of maltreatment, and physical neglect (3.0%) was least reported; however, among those who only reported one maltreatment type, sexual abuse was most common (Additional file 2: Fig. S1). Participants who reported any maltreatment were more likely to be female, younger, non-white, and born outside of the UK and to have a higher number of siblings, a mother who smoked around birth, and a mother with severe depression. Participants who reported no maltreatment were more likely to be breastfed as a baby. Compared to the rest of the UK Biobank sample, participants with maltreatment data were slightly more likely to be female, white, and born in the UK (Additional file 1: Table S4).

### Observational outcome-wide analysis

**Fig. 2:**
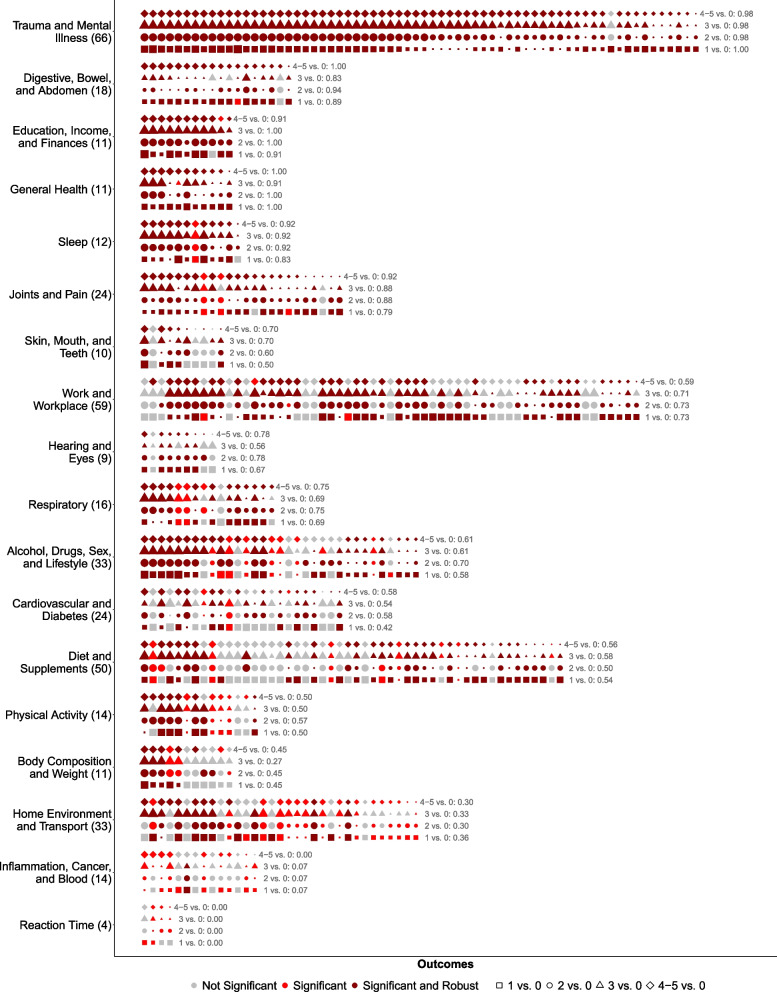
Observational outcome-wide analysis results by theme. Statistical significance and relative effect sizes from the observational OWA for maltreatment count 4–5 vs. 0 (diamonds), 3 vs. 0 (triangles), 2 vs. 0 (circles), and 1 vs. 0 (squares), grouped by theme. Statistical significance is assessed after false discovery rate correction at *α* = 0.05. Results are further categorized as robust according to the *E*-value metric; see the “Methods” section for details. The size of each shape reflects the strength of the association, with the largest shapes reflecting associations in the top decile and the smallest shapes reflecting the lowest decile of effect sizes across beta and OR/RR estimates. Themes are sorted (top to bottom) by the proportion of results within each theme that were considered significant and robust, according to the test of trend. Within each theme, results are sorted (left to right) by the relative effect size from the maltreatment count 4–5 vs. 0. Number of total results per theme is listed in parentheses on the *y*-axis. Proportion of results that are significant and robust per theme and maltreatment count comparison are listed in gray text

We observed statistically significant and *robust* relationships between childhood maltreatment and two-thirds of outcomes when comparing both 4–5 types and 1 type of maltreatment to none, capturing the profound impact of *any* maltreatment. We present primary results for each outcome in Fig. 2 and Additional file 1: Table S5, grouping outcomes into the 18 broad themes for clarity. Results from primary analyses and sensitivity analyses that used the continuous maltreatment variable were consistent (Additional file 1: Table S6). Therefore, for brevity and interpretability, we further discuss only the test of trend results. According to the test of trend, more than 70% of outcomes within each of the following ten themes had both significant tests of trend and robust effects: digestive, bowel, and abdomen; education, income, and finances; general health; hearing and eyes; joints and pain; respiratory; skin, mouth, and teeth; sleep; trauma and mental illness; and work and workplace (Additional file 1: Table S7). Many additional significant and robust associations (> 50% of outcomes) were observed within the domains of alcohol, drugs, sex, and lifestyle; cardiovascular and diabetes; diet and supplements; and physical activity. Few significant and robust associations (< 50% of outcomes) were observed among outcomes related to body composition and weight; home environment and transport; inflammation, cancer, and blood; and reaction time. Overall, the strongest relative effect sizes, as displayed in Fig. 2, came from the domains of education, income, and finances; trauma and mental illness; sleep; work and workplace; and general health.

Sex-stratified analyses were largely similar to unstratified results, apart from two outcomes (Additional file 2: Fig. S2A and Additional file 1: Tables S8–S9). According to the test of trend, increasing maltreatment count was associated with greater odds of ever being employed as a director or chief executive and of omega-3 supplement use for women, whereas the reverse was true for men.

### Genetic outcome-wide analysis

**Fig. 3:**
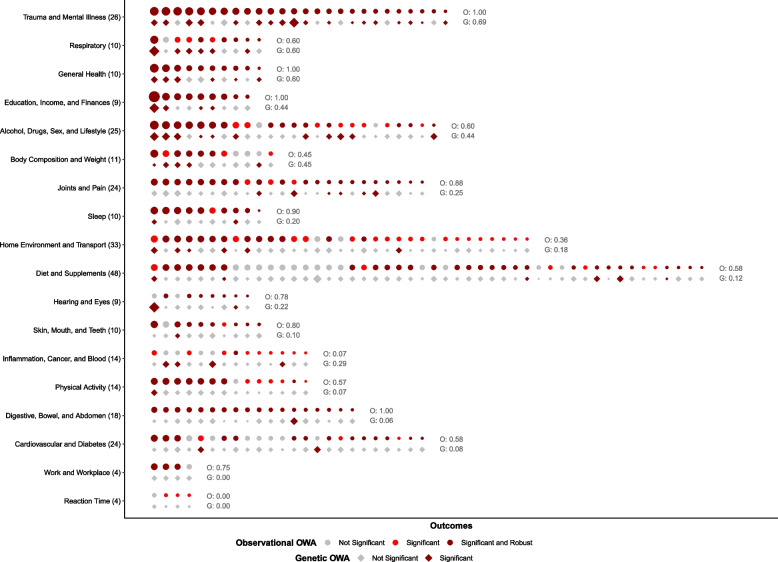
Triangulation of results across observational and genetic outcome-wide analyses by theme. Statistical significance and relative effect sizes from the observational OWA (circles) and genetic OWA (diamonds), grouped by theme. For each OWA, statistical significance is assessed after false discovery rate correction at *α* = 0.05. For the observational OWA, results are further categorized as robust according to the *E*-value metric; see the “Methods” section for details. For each OWA, the size of each shape reflects relative strength of the association across beta and OR/RR estimates. Themes are sorted (top to bottom) by the proportion of results within each theme that were considered significant in both OWAs. Within each theme, results are sorted (left to right) by the relative effect size from the observational OWA. Number of total results per theme is listed in parentheses on the *y*-axis. Proportion of results that are significant (and robust, for the observational OWA) per theme and OWA are listed in grey text

In total, we observed statistically significant relationships between the PRS comprised of genome-wide significant SNPs and 26.7% of all outcomes examined (Fig. 3, Additional file 1: Table S10). More than 40% of outcomes within each of the following themes had significant results: alcohol, drugs, sex, and lifestyle; body composition and weight; education, income, and finances; general health; respiratory; and trauma and mental illness. Some additional significant associations (> 10% of outcomes) were observed within the domains of diet and supplements; hearing and eyes; home environment and transport; inflammation, cancer, and blood; joints and pain; skin, mouth, and teeth; and sleep. Few significant associations (< 10% of outcomes) were observed within the themes of cardiovascular and diabetes; digestive, bowel, and abdomen; physical activity; reaction time; or work and workplace. Overall, the strongest relative effect sizes, as visualized in Fig. 3, came from the domains of hearing and eyes; respiratory; trauma and mental illness; education, income, and finances; and alcohol, drugs, sex, and lifestyle.

Analyses with PRS derived from a *p*-value threshold of 0.5 were consistent with the genome-wide significant PRS results but captured more signals across our outcomes (Additional file 1: Table S11 and Additional file 2: Fig. S3). This finding was expected, given that the PRS with a greater number of SNPs explained more phenotypic variance in hold-out sample analyses by Warrier and colleagues [56] but was also likely more vulnerable to noise and instrumental variable violations [86–89]. Results from models using PRS quintiles were largely similar, as were sex-stratified results (Additional file 2: Fig. S2B and Additional file 1: Tables S12–S13).

### Triangulation

**Fig. 4:**
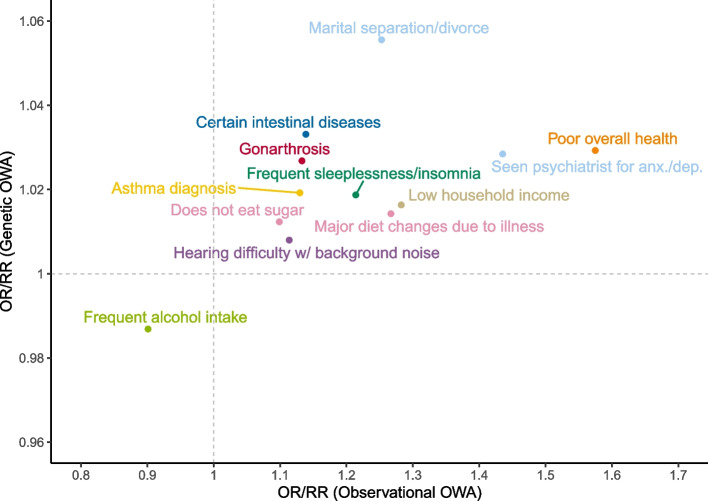
Scatterplot of effect estimates from a subset of concordant results across two outcome-wide analyses. Effect estimates for a subset of concordant results discussed in the “Results” section. The OR/RR from the observational OWA is plotted along the *x*-axis, and the OR/RR from the genetic OWA is plotted along the *y*-axis. Color reflects the broader health theme to which each outcome belongs

We triangulated results using the continuous maltreatment variable and the standardized PRS at genome-wide significance. Recall that in the genetic OWA, the associations are with the polygenic risk score rather than maltreatment itself; for simplicity, however, we refer broadly to maltreatment exposure in this section. Of the 303 maltreatment-outcome associations estimated in the observational and genetic OWAs, 25% were significant in both analyses and concordant in the direction of association (Fig. 3). Of those concordant results, 83% were considered robust in the observational OWA based on *E*-value assessment. Within the latter set of results, maltreatment was associated with poor overall health (OR from observational OWA, OR_*o*_ = 1.57 (95% CI: 1.53, 1.62); *E*-value from observational OWA, *E*_*o*_ = 2.43; OR from genetic OWA, OR_*g*_ = 1.03 (1.01, 1.05)). Among behavioral and relational factors, maltreatment was associated with a higher lifetime number of sexual partners (*β*_*o*_ = 0.13 (0.12, 0.14); *E*_*o*_ = 1.49; *β*_*g*_ = 0.02 (0.01, 0.02)) and higher odds of both marital separation/divorce (OR_*o*_ = 1.25 (1.21, 1.29); *E*_*o*_ = 1.72; OR_*g*_ = 1.06 (1.03, 1.08)) and major diet changes due to illness (OR_*o*_ = 1.27 (1.24, 1.29); *E*_*o*_ = 1.79; OR_*g*_ = 1.01 (1.00, 1.03)), but lower odds of frequent weekly alcohol intake (OR_*o*_ = 0.90 (0.89, 0.91); *E*_*o*_ = 1.42; OR_*g*_ = 0.99 (0.98, 0.99)) and higher odds of not eating sugar (OR_*o*_ = 1.10 (1.08, 1.11); *E*_*o*_ = 1.38; OR_*g*_ = 1.01 (1.00, 1.02)). Across domains of physical health, we identified an association with higher BMI (*β*_*o*_ = 0.09 (0.08, 0.09); *E*_*o*_ = 1.38; *β*_*g*_ = 0.01 (0.00, 0.01)) in addition to higher odds of certain intestinal diseases (OR_*o*_ = 1.14 (1.10, 1.18); *E*_*o*_ = 1.42; OR_*g*_ = 1.03 (1.01, 1.06)), hearing difficulty with background noise (OR_*o*_ = 1.11 (1.11, 1.12); *E*_*o*_ = 1.45; OR_*g*_ = 1.01 (1.00, 1.01)), gonarthrosis (knee arthrosis; OR_*o*_ = 1.13 (1.09, 1.18); *E*_*o*_ = 1.41; OR_*g*_ = 1.03 (1.01, 1.05)), and asthma diagnosis (OR_*o*_ = 1.13 (1.11, 1.15); *E*_*o*_ = 1.46; OR_*g*_ = 1.02 (1.01, 1.03)). Finally, maltreatment was associated with numerous mental health-related outcomes, including higher odds of frequent sleeplessness/insomnia (OR_*o*_ = 1.21 (1.20, 1.23); *E*_*o*_ = 1.68; OR_*g*_ = 1.02 (1.01, 1.03)) and seeing a psychiatrist for anxiety or depression (OR_*o*_ = 1.44 (1.42, 1.45); *E*_*o*_ = 2.18; OR_*g*_ = 1.03 (1.02, 1.04)), and socioeconomic indicators, including higher odds of low household income (OR_*o*_ = 1.28 (1.26, 1.31); *E*_*o*_ = 1.83; OR_*g*_ = 1.02 (1.01, 1.03)). The subset of concordant results discussed here are plotted in Fig. 4 (binary/categorical outcomes only). While some results from the cardiovascular, inflammation, reaction time, and workplace domains were statistically significant across OWAs, none were considered robust in the observational OWA.

Of all 303 associations estimated, approximately 62% were significant in the observational OWA but not the genetic OWA. Of those, 74% were considered robust in the observational OWA. Significant and robust relationships unique to the observational OWA included numerous cardiovascular outcomes, such as heart attack (ICD: OR_*o*_ = 1.15 (1.09, 1.22); *E*_*o*_ = 1.40; self-reported diagnosis: OR_*o*_ = 1.09 (1.08, 1.10); *E*_*o*_ = 1.36), chronic ischemic heart disease (ICD: OR_*o*_ = 1.18 (1.14, 1.23); *E*_*o*_ = 1.53), and high blood pressure (self-reported: OR_*o*_ = 1.30 (1.24, 1.36); *E*_*o*_ = 1.79). Others included gastro-esophageal reflux disease (ICD: OR_*o*_ = 1.17 (1.13, 1.21); *E*_*o*_ = 1.50; self-reported: OR_*o*_ = 1.14 (1.10, 1.17); *E*_*o*_ = 1.43), education (college/university degree: OR_*o*_ = 0.95 (0.94, 0.96); *E*_*o*_ = 1.26), cataracts (ICD: OR_*o*_ = 1.06–1.12, depending on type; self-reported: OR_*o*_ = 1.15 (1.09, 1.21); *E*_*o*_ = 1.40), migraine (self-reported: OR_*o*_ = 1.10 (1.06, 1.13); *E*_*o*_ = 1.31), cancer (self-reported: OR_*o*_ = 1.07 (1.04, 1.10); *E*_*o*_ = 1.26), and mouth ulcers (self-reported: OR_*o*_ = 1.10 (1.08, 1.12); *E*_*o*_ = 1.37$$)$$.

Six outcomes were significantly associated in the genetic OWA and null in the observational OWA, including diastolic blood pressure (*β*_*g*_ =  − 0.01 (− 0.01, − 0.01)$$)$$ and glaucoma (OR_*g*_ = 0.94 (0.90, 0.98)). No outcomes were statistically significant in opposite directions in the two analyses. Finally, 11% of all outcomes were not significantly associated with maltreatment in either OWA. Over half of these involved medication use or dietary patterns; others included atrial fibrillation/flutter and cognitive function. Additional file 2: Fig. S4 provides an overview of concordance, and Additional file 1: Table S6 summarizes results by theme and analytic method.

## Discussion

We conducted two large-scale OWAs that examined the relationship between childhood maltreatment and hundreds of outcomes capturing health and well-being in adulthood. First, we triangulated observational evidence with that of a genetic OWA and identified robust associations of maltreatment with increased risk of mental illness, insomnia, health risk behaviors, asthma, pain, high BMI, and low socioeconomic status, among other outcomes. Second, we linked self-reported maltreatment to a range of previously underexamined outcomes, including a higher risk of hearing difficulties, blurred vision, dental problems, and digestive diseases. Third, many of the novel associations identified in our observational OWA were unlikely to be explained by expected levels of unmeasured confounding, as quantified in sensitivity analyses using *E*-values [51–53]. Altogether, our results highlight the far-reaching negative effects of childhood maltreatment in later life, which include both leading causes of mortality as well as extensive influences on quality of life.

In the present study, our concordant results largely aligned with existing literature. We triangulated results linking maltreatment to poorer outcomes across the domains of mental illness [10, 12], sleep disorders [90, 91], chronic pain [40], chronic lung diseases [8], risk behaviors [92–95], and socioeconomic status [19]. This outcome-wide study does not allow for the investigation of mechanisms underlying these associations. Prior evidence suggests that psychological (e.g., post-traumatic stress disorder), behavioral (e.g., physical activity), and biological (e.g., immune dysregulation) pathways are likely at play [96–100].

We also identified results unique to each OWA. While our observational OWA identified relationships between maltreatment and many outcomes within the domains of ocular and oral health, digestive diseases, cardiovascular diseases, and related risk factors such as diet and physical activity, the genetic OWA for the most part did not identify such associations. On the other hand, the genetic OWA uniquely identified significant relationships of maltreatment PRS with diastolic blood pressure, glaucoma, and various white blood cell measures. Discordant results may reflect biases resulting in spurious associations or limitations preventing the detection of a true association in only one of the two OWAs. For example, the signals identified only in the observational OWA may be inflated due to residual confounding by childhood socioeconomic status that was not fully captured by maternal smoking status, given the association of childhood socioeconomic status with diet [101, 102], physical activity [103], cardiovascular disease [104, 105], and digestive diseases [106] in adulthood. However, compared to the maternal smoking at birth coefficient, our *E*-values for many of the results unique to the observational OWA were large, indicating plausible robustness to residual confounding by childhood socioeconomic status. Specifically, prior literature has demonstrated a strong association between maternal smoking during pregnancy and socioeconomic status (e.g., up to sixfold increase in smoking among the lowest vs. highest educated groups) [83, 84], making it less likely for an unmeasured socioeconomic confounder to correlate as strongly with *both* maltreatment [107] and the outcome in question as did maternal smoking status, *beyond* concurrent adjustment for maternal smoking status. True effect sizes between maltreatment and outcomes related to digestive and cardiovascular diseases, among others, may be smaller than what was reported in the observational OWA due to residual confounding but likely still non-zero. Furthermore, our PRS at genome-wide significance was likely underpowered [56], and secondary results using PRS based on a p-value threshold of 0.5 exhibited a higher number of significant associations in these domains. While the higher proportion of significant results from the larger PRS should be interpreted with additional caution given the vulnerability of a PRS with more SNPs to noise and violations of instrumental variable assumptions [56, 86–89], results across observational and genetic OWAs may not be as discordant as they appear. As postulated in prior studies that also found inconsistent results linking maltreatment to cardiovascular outcomes [34–36], discordant results for blood pressure may be explained by the limitations of our blood pressure measures, the differing relationship of maltreatment with point-in-time measurements versus trajectories of blood pressure, and our inability to account for antihypertensive medication use within the outcome-wide framework. Future observational studies should triangulate across different sources of data, as we explored with blood pressure (self-reported diagnosis vs. laboratory point-in-time measures of blood pressure); future genetic studies may look to identify a higher-powered PRS to further maltreatment investigations.

Our study has several strengths. First, we prioritized an agnostic, data-driven approach to outcome selection, leveraging prior applications of data reduction techniques to distill large-scale phenotype data [66]. In doing so, we examined a comprehensive set of outcomes that contribute meaningfully to both the variation and correlation structures in the human phenome, allowing for comparison of the impact of maltreatment across a large range of relevant outcomes while limiting investigator influence on the outcome selection process. Several outcomes in our comprehensive list have received scant prior attention. Second, we used *E*-values to examine the robustness of our observational results to unmeasured confounding [83]. Comparison of the *E*-value and the maternal smoking coefficient, though not immune to bias or violation of assumptions [51], provided a useful metric for determining potential robustness of results. Third, we triangulated results from observational and genetic OWAs with different biases, demonstrating how this outcome-wide triangulation design may be applied to strengthen both novel discovery and causal inference. The results from the observational OWA may be inflated due to confounding by environmental factors [19, 46, 47] or mood-dependent recall bias [44, 45], whereby individuals with a higher burden of mental illness are more likely to report adverse experiences during childhood. The latter phenomenon may have particularly inflated results from the mental health domain. In contrast, the PRS for maltreatment relies on genetic variants that are assigned independently at conception and thus should be less susceptible to environmental confounding; furthermore, the high correlation (*r*_*g*_ = 0.72) between GWAS for prospectively and retrospectively assessed childhood maltreatment indicates that the PRS we used based on a meta-GWAS of retrospectively and prospectively assessed childhood maltreatment was unlikely to be affected by recall bias [54, 56]. Conversely, the genetic OWA is limited by the low variance explained by the PRS [56], resulting in weak instrument bias towards the null [55, 108, 109], and may also be subject to residual population stratification [108] and horizontal pleiotropy (a direct effect of genetics on the outcome that does not act through maltreatment) [109, 110]. However, the orthogonal features of these approaches allowed us to triangulate concordant results for more compelling evidence of maltreatment’s profound impact on health.

In our study, we used the PRS for childhood maltreatment as a tool in the context of outcome-wide analysis to strengthen evidence for the causal adverse effects of childhood maltreatment on a wide range of health domains. The PRS used in this study was developed by Warrier et al., who found that childhood maltreatment is moderately heritable and emerges through the complex interplay of genetic and environmental factors not yet fully understood [56]. Additionally, the heritability of childhood maltreatment is likely at least partially explained by intergenerational transmission, whereby both genes and environments are passed down from parents to children. As noted by Warrier et al., the heritability of childhood maltreatment does “not imply that environmental factors are absent, that the child is to blame, or that the heritability is fixed” ([56] p. 383). We would add that the heritability of childhood maltreatment does not say anything about the effectiveness of environmental interventions to prevent childhood maltreatment or its downstream adverse effects on health. Meta-analysis suggests specific components of interventions (e.g., parenting skills) that are effective in both preventing and reducing childhood maltreatment and ameliorating its adverse effects [111, 112]. For a detailed discussion of the broader implications of research on genetic influences on childhood maltreatment, see the Appendix from Warrier et al. [56].

At least seven study limitations should also be considered. First, in the observational OWA, we relied on retrospective self-reports of maltreatment based on a brief screener and were not able to examine the concordance of these reports with prospective observations of maltreatment. Researchers have documented poor agreement between prospective and retrospective measures of childhood maltreatment and between self-reports and reports from other informants [48, 49]. Of key concern is the role of memory biases related to mood and psychopathology at the time of maltreatment reporting, as mentioned above [44, 45]. It is possible that some of the associations identified in our observational OWA would not replicate in an analysis that used prospective observations of maltreatment [50]. This limitation is less of a concern with our genetic instrument, given the strong genetic correlation between retrospective and prospective reports of maltreatment (*r*_*g*_ = 0.72) [56]. Second, the UK Biobank has limited information on paternal factors from early childhood and thus we could not adjust for paternal factors such as age and lifestyle that may confound the association between maltreatment and health outcomes [113–117]. Third, the UK Biobank sample is healthier and wealthier than the UK population, which may impact the external validity of our results [71]. Fourth, we cannot rule out confounding of the PRS-health relationships by other phenotypes that share genetic architecture with maltreatment in a way that may bias results, depending on the pathways involved. This limitation weakens the argument of orthogonal bias between the two OWAs and should be robustly investigated in extensions of this work. Fifth, the GWAS used to inform our PRS excluded individuals of non-European ancestry [56], and due to the considerably lower accuracy of PRS in non-European ancestry individuals when drawing on Eurocentric GWAS [69, 70], we were unable to include such groups in our genetic OWA. Our group [118–120] and others [121–126] are working to expand genetic studies in non-European ancestry populations, which have been sorely underrepresented in such work. Given the higher burden of adversity (including maltreatment and many of the outcomes investigated here) in such underrepresented populations [127–133], expansion of this current work is critical. Sixth, certain outcomes were omitted, as variables that were systematically missing were excluded from the prior factor analysis; these included reproductive and maternal health outcomes, previously linked to maltreatment, that were only asked of female participants [66, 134, 135]. Finally, despite the large sample, specific analyses had limited power due to low outcome prevalence, and we were not able to examine the effects of different types of maltreatment.

Despite these limitations, multiple clinical and methodological implications emerge from our findings. We demonstrated the utility of an outcome-wide triangulation design with sensitivity analyses to examine the wide-ranging effects of childhood maltreatment on health and well-being in later life and to address the pervasive challenges of confounding and outcome selection in the field of maltreatment research. Future studies may extend our research using other causal approaches such as the numerous methods of Mendelian Randomization or genomic SEM, which would allow for a more thorough examination of potential biases related to horizontal pleiotropy and shared genetic architecture of complex traits [54, 136–138]. Studies with comprehensive longitudinal data may extend these analyses with longitudinal or time-to-event analyses and may investigate relationships between outcomes and the likely presence of mediators and moderators in the pathways linking maltreatment and health [139]. From a clinical perspective, screening for childhood maltreatment may be an effective tool for identifying those at increased risk of adverse outcomes, given the diverse potential consequences of childhood maltreatment on human health and well-being identified. Though research on routine screening for adverse childhood experiences remains limited [140], the expected individual- and population-level benefits of mitigating the health consequences of childhood maltreatment are considerable [141]. Additionally, the prevalence of maltreatment and its relationship with myriad domains of health and functioning–including those previously understudied–reinforces the importance of trauma-informed and integrated healthcare across specialties [142, 143]. In conclusion, by utilizing big data, genetics, and the outcome-wide framework, we underscore the urgent need to intervene upon the sweeping effects of childhood maltreatment on long-term health and well-being.

## Conclusions

Using an outcome-wide triangulation design with sensitivity analyses, we investigated the relationship of childhood maltreatment with over 400 health outcomes in later life. Our study design proved useful for both novel discovery and causal inference, drawing on orthogonal features of leading epidemiologic approaches to document the far-reaching negative effects of maltreatment on both leading causes of mortality and diverse markers of quality of life.

### Supplementary Information

**Additional file 1.** Supplemental tables. **Table S1.** Coding of Five Types of Childhood Maltreatment from the Childhood Trauma Screener Validation Study [74]. **Table S2.** List of Study Outcomes Based on Carey et al. (2022) Factor Analysis Loadings [66]. **Table S3A.** List of Non-Outcome Study Variables Included in Multiple Imputation Model. **Table S3B.** Distribution of 'Prefer Not to Say' Responses Across Maltreatment Types by Number of 'Prefer Not to Say' Responses. **Table S3C.** Distribution of Responses to Maltreatment Questions Before and After Imputation. **Table S4.** Comparison of UK Biobank Mental Health Questionnaire Respondents and Non-Respondents. **Table S5.** Observational Outcome-Wide Analysis of Childhood Maltreatment Categorical Count on Outcomes in Adulthood. **Table S6.** Observational Outcome-Wide Analysis of Childhood Maltreatment Trend Test on Health Outcomes in Adulthood. **Table S7.** Summary of Significance of Results by Health Theme for Outcome-Wide Analyses (Overall and Sex-Stratified). **Table S8.** Observational Outcome-Wide Analysis of Childhood Maltreatment on Health Outcomes in Adulthood (Females Only). **Table S9.** Observational Outcome-Wide Analysis of Childhood Maltreatment on Health Outcomes in Adulthood (Males Only). **Table S10.** Genetic Outcome-Wide Analysis of Childhood Maltreatment Polygenic Risk Score (*P*-Value Threshold 5e-08) on Outcomes in Adulthood. **Table S11.** Genetic Outcome-Wide Analysis of Childhood Maltreatment Polygenic Risk Score (*P*-Value Threshold 0.5) on Health Outcomes in Adulthood. **Table S12.** Genetic Outcome-Wide Analysis of Childhood Maltreatment Polygenic Risk Score on Health Outcomes in Adulthood (Females Only). **Table S13.** Genetic Outcome-Wide Analysis of Childhood Maltreatment Polygenic Risk Score on Health Outcomes in Adulthood (Males Only).

**Additional file 2.** Supplemental figures. **Figure S1.** Childhood Maltreatment Set-Intersections Plot. **Figure S2.** Results Triangulation Across Sex-Stratified Outcome-Wide Analyses. **Figure S3.** Genetic Outcome-Wide Analysis Results by Theme. **Figure S4.** Results Concordance Across Observational and Genetic Outcome-Wide Analyses.

## Data Availability

Genetic and phenotypic UK Biobank data are available through an application procedure described at https://www.ukbiobank.ac.uk/enable-your-research. Summary statistics from the meta-GWAS of childhood maltreatment are available at 10.17863/CAM.65339 [68]. We did not pre-register our analysis plan or hypotheses.

## Abbreviations

FDR: False discovery rate
HWE: Hardy-Weinberg Equilibrium
MAF: Minor allele frequency
OWA: Outcome-wide analysis
PRS: Polygenic risk score
QC: Quality control
SNP: Single nucleotide polymorphism
