# Supplementary material for: Childhood maltreatment and health in the UK Biobank: triangulation of outcome-wide and polygenic risk score analyses
Source: BMC Med. 2024 Mar 25;22:135. doi: 10.1186/s12916-024-03360-9 (PMC10962116; doi:10.1186/s12916-024-03360-9)
Supplement: Supplementary file 2 — Additional file 2. Supplemental figures. Figure S1. Childhood Maltreatment Set-Intersections Plot. Figure S2. Results Triangulation Across Sex-Stratified Outcome-Wide Analyses. Figure S3. Genetic Outcome-Wide Analysis Results by Theme. Figure S4. Results Concordance Across Observational and Genetic Outcome-Wide Analyses. [file 12916_2024_3360_MOESM2_ESM.docx]

**Figure S1: Childhood Maltreatment Set-Intersections Plot**

Top 10 most common childhood maltreatment type set intersections prior to imputation in analytic sample used for the observational OWA. Plot is made up of 5 rows (one row per maltreatment type) and 10 columns (top 10 set intersections) that combine to describe both the prevalence of exposure to each maltreatment type (regardless of exposure to other types; left plot) and the prevalence of exposure to a certain combination of maltreatment types (right plot; e.g., sexual abuse but no other abuse or neglect).

**Figure S2: Results Triangulation Across Sex-Stratified Outcome-Wide Analyses**

For each outcome-wide analysis (OWA), statistical significance (“sig” if significant, “null” otherwise) is assessed after false discovery rate correction at α=0.05. Sex-stratified results are compared based on both significance and direction of association. There are 419 results for 414 outcomes from the observational OWA due to separate reporting of each coefficient for the four categorical variables. For the same reason, there are 303 results for 298 outcomes from the genetic OWA. Observational OWA includes 89,073 females and 68,243 males; genetic OWA includes 127,991 females and 115,015 males.

**Figure S3: Genetic Outcome-Wide Analysis Results by Theme**

Statistical significance and relative effect sizes from the genetic OWA for PRS at p-value threshold 0.5 (circles) and genome-wide significance (diamonds), grouped by theme. Statistical significance is assessed after false discovery rate correction at α=0.05. For each PRS analysis, the size of each shape reflects strength of the association, with largest shapes reflecting associations in the top decile and smallest shapes reflecting lowest decile of effect sizes across beta and OR/RR estimates. Themes are sorted (top to bottom) by the proportion of results within each theme that are considered significant, according to the genetic OWA for PRS at genome-wide significance. Within each theme, results are ordered (left to right) by the relative effect size from the PRS at p-value threshold 0.5. Number of total results per theme is listed in parentheses on the y-axis. Proportion of results that are significant per theme and p-value threshold are listed in grey text.

**Figure S4: Results Concordance Across Observational and Genetic Outcome-Wide Analyses**

For each outcome-wide analysis (OWA), statistical significance is assessed after false discovery rate correction at α=0.05. There are 419 results from the observational OWA for 414 outcomes from the observational OWA due to separate reporting of each coefficient for the four categorical variables. For the same reason, there are 303 results for 298 outcomes from the genetic OWA.

A. For the observational OWA, results are further categorized as robust according to the E-value metric; see Methods section for details.

B. For the genetic OWA, outcomes from the observational OWA that were not included are omitted from this graph.

C. We compared observational and genetic OWA results based on both significance (“sig” if significant; “null” otherwise) and direction of association.
